# Supplementary material for: Quality assessment from biobank plasma and serum specimens: a systematic review
Source: PeerJ. 2025 Oct 8;13:e20122. doi: 10.7717/peerj.20122 (PMC12514999; doi:10.7717/peerj.20122)
Supplement: Supplemental Information 3 [file peerj-13-20122-s003.docx]

Systematic Review Rationale

1. **Fundamental Principles**

Biobanks are essential infrastructures for translational medicine and precision healthcare, with the quality of their samples directly influencing the reliability of research data and the effectiveness of clinical translation. However, plasma and serum samples face complex pre-analytical variables (such as delays in processing, freeze-thaw cycles, and storage conditions) during collection, processing, storage, and pre-analysis. These factors can lead to biomarker degradation or variability, thereby affecting the reproducibility of research results. Although international guidelines (e.g., ISO standards) and best practices (e.g., the SPREC framework) have provided some recommendations, the degree of standardization across biobanks remains inconsistent. Moreover, there is a lack of a systematic framework specifically designed for assessing the quality of plasma and serum samples. Therefore, there is an urgent need to systematically integrate existing evidence, identify key influencing factors, and establish a multidimensional evaluation system. Such efforts would optimize biobank management, improve sample quality, and enhance research credibility.

**2. Contribution to Existing Knowledge**

To date, no systematic review has specifically addressed the quality assessment of plasma and serum samples. Prior systematic reviews, such as the one by Caixeiro et al. (2016) on RNA quality in biobank tissue samples, have highlighted that reliance on a single quality metric (e.g., RNA Integrity Number, RIN) fails to provide a comprehensive evaluation of sample quality(Caixeiro et al. 2016). Furthermore, the lack of standardization of pre-analytical variables (such as storage time and temperature) continues to be a central challenge in biobank management. This research emphasizes the need for a multidimensional assessment combining both diagnostic and predictive indicators. However, it primarily focuses on RNA quality in tissue samples and does not address the unique issues associated with plasma and serum liquid samples. Other reviews, while addressing biobank quality control, tend to focus on specific biomarkers or technical methods (such as ELISA or mass spectrometry) and lack a comprehensive analysis of the systematic quality assessment of plasma and serum samples. This review integrates 48 studies and systematically explores the impact of pre-analytical variables (such as processing delays, freeze-thaw cycles, and storage conditions) on biomarker stability in plasma and serum samples. It also identifies gaps in existing research, such as the neglect of pre-collection factors like medication use and dietary habits, thus filling a critical gap in the field of liquid sample quality assessment.

**3. Objective and Significance of This Review**

This review represents the first comprehensive effort to synthesize evidence in this area, revealing the limitations of current quality assessment methods (such as reliance on single parameters and lack of standardization) and proposing a multi-biomarker assessment strategy. By analyzing the stability of biomarkers such as enzymes, cytokines, and metabolites under varying conditions, this study provides scientific evidence to support the development of biobank operational standards. Additionally, the review highlights the potential of integrating artificial intelligence and multi-omics technologies to optimize quality assessment, pointing the way forward for future research. Ultimately, the goal of this review is to promote the standardization of biobank sample management, enhance the reproducibility of research data, and lay the foundation for high-quality samples in the clinical translation of precision medicine.

Caixeiro NJ, Lai K, and Lee CS. 2016. Quality assessment and preservation of RNA from biobank tissue specimens: a systematic review. *Journal of clinical pathology* 69:260-265. 10.1136/jclinpath-2015-203384
